# Human Health Risk Assessment of Artisanal Miners Exposed to Toxic Chemicals in Water and Sediments in the Prestea Huni Valley District of Ghana

**DOI:** 10.3390/ijerph13010139

**Published:** 2016-01-18

**Authors:** Samuel Obiri, Philip O. Yeboah, Shiloh Osae, Sam Adu-kumi, Samuel J. Cobbina, Frederick A. Armah, Benjamin Ason, Edward Antwi, Reginald Quansah

**Affiliations:** 1 Department of Nuclear and Environmental Protection, School of Nuclear and Allied Sciences, Ghana Atomic Energy Commission, P.O. Box AE 1, Atomic, Accra, Ghana; poyeboah47@yahoo.co.uk (P.O.Y.); s.osae@gaecgh.org (S.O.); 2 Centre for Environmental Impact Analysis, P.O. Box AD 738, Cape Coast, Ghana; 3 Environmental Protection Agency, P.O. Box M. 326, Accra, Ghana; adukumisam@yahoo.com; 4 Department of Ecotourism and Environmental Management, Faculty of Renewable Natural Resources, University for Development Studies, P.O. Box TL 1882, Nyankpala Campus, Tamale, Ghana; cobbinasamuel@yahoo.com; 5 Department of Environmental Science, University of Cape Coast, Cape Coast, Ghana; farmah@ucc.edu.gh; 6 CSIR—Soil Research Institute, P.O. Box M. 32, Accra, Ghana; soilresearchben@gmail.com; 7 Department of Chemical Engineering, KNUST, Kumasi, Ghana; edwardantwi@hotmail.com; 8 Department of Immunology, Noguchi Memorial Institute for Medical Research, College of Health and Allied Sciences, University of Ghana, Legon, Ghana; yaw121@yahoo.co.uk

**Keywords:** non-cancer risk, cancer health risk, disease profile, PresteaHuni Valley, hazard quotient

## Abstract

A human health risk assessment of artisanal miners exposed to toxic metals in water bodies and sediments in the PresteaHuni Valley District of Ghana was carried out in this study, in line with US EPA risk assessment guidelines. A total of 70 water and 30 sediment samples were collected from surface water bodies in areas impacted by the operations of artisanal small-scale gold mines in the study area and analyzed for physico-chemical parameters such as pH, TDS, conductivity, turbidity as well as metals and metalloids such as As, Cd, Hg and Pb at CSIR—Water Research Institute using standard methods for the examination of wastewater as outlined by American Water Works Association (AWWA). The mean concentrations of As, Cd, Hg and Pb in water samples ranged from 15 μg/L to 325 μg/L (As), 0.17 μg/L to 340 μg/L (Cd), 0.17 μg/L to 122 μg/L (Pb) and 132 μg/L to 866 μg/L (Hg), respectively. These measured concentrations of arsenic (As), mercury (Hg), cadmium (Cd) and lead (Pb) were used as input parameters to calculate the cancer and non-cancer health risks from exposure to these metals in surface water bodies and sediments based on an occupational exposure scenario using central tendency exposure (CTE) and reasonable maximum exposure (RME) parameters. The results of the non-cancer human health risk assessment for small-scale miners working around river Anikoko expressed in terms of hazard quotients based on CTE parameters are as follows: 0.04 (Cd), 1.45 (Pb), 4.60 (Hg) and 1.98 (As); while cancer health risk faced by ASGM miners in Dumase exposed to As in River Mansi via oral ingestion of water is 3.1 × 10^−3^. The hazard quotient results obtained from this study in most cases were above the HQ guidance value of 1.0, furthermore the cancer health risk results were found to be higher than the USEPA guidance value of 1 × 10^−4^ to 1 × 10^−6^. These findings call for case-control epidemiological studies to establish the relationship between exposure to the aforementioned toxic chemicals and diseases associated with them as identified in other studies conducted in different countries as basis for developing policy interventions to address the issue of ASGM mine workers safety in Ghana.

## 1. Introduction

Gold mining in Ghana has played a substantive role in the socio-economic and political life of indigenes for millennia [1,2]. The sector is made up of small-scale (artisanal) mining and large-scale mining. Artisanal miners usually include unemployed indigenous youth who have little financial backing and limited mining expertise, but who have acquired the requisite mining license to operate. Another group within the artisanal small-scale sector consists of individuals and groups engaged in illegal mining activity known locally as “*galamsey*” [3]; that is, the practice of discreetly gathering minerals found either at or just below the soil surface and selling them in contravention of national laws [4]. In 1986 the nation formally allowed its citizens to operate as artisanal small-scale miners; since that time, this sector has employed over 200,000 people [3,5,6,7,8,9,10,11].

Metalloids such as arsenic (As) and heavy metals such as lead (Pb), mercury (Hg) and cadmium (Cd) are well-studied, naturally occurring pollutants whose environmental levels have increased in artisanal mining communities [12,13,14]. For instance, mercury pollution has been identified as a lingering problem in several of Ghana’s important artisanal gold mining communities. A burgeoning collection of studies (e.g., [14,15]) have demonstrated that contamination is now widespread in biota, with serious health implications for human populations residing within the country’s gold belts.

Concerns from exposure to environmental pollutants have led to an increase in the measures taken to reduce the release of these chemicals into the environment and to protect miners and the general public. ASGM miners in the study area either work on 8 h shift basis (based on Central Tendency Exposure parameters) or 12 h a day (based Reasonable Maximum Exposure). Their activities usually involve dredging of river beds for alluvial gold or washing of the gold ores blocked rivers and in the process incidentally ingest both the contaminated water and the sediment or through dermal exposure to the contaminants.

However, very little work has been done in assessing the human health risk associated with occupational (*i.e.*, exposure to toxic chemicals due to one’s work schedule) exposure to toxic metals by artisanal gold miners in the Prestea Huni Valley District in the Western Region of Ghana. It is against this background that this study was carried out. The default exposure values and the assumptions governing their use in this study is presented in Table 1 under Section 2.5.3 calculation of carcinogenic and non-carcinogenic health risk.

The main objectives of this study were: (1) to determine the concentrations of As, Pb, Cd, and Hg in water bodies and sediments in the study area; (2) to evaluate the potential cancer and non-cancer human health risk to ASGM miners associated with exposure to these metals through ingestion of, and dermal contact with, water and sediment in the study area; and (3) compare the cancer and non-cancer health risk results of this study with published cancer and non-cancer health risk guidance values.

This paper therefore contributes to a growing literature on the human health impacts associated with exposure to toxicants by artisanal miners in developing countries. It may serve as the basis of assessment that will inform policy-makers in Ghana, other developing countries, and international donor communities as they develop and implement appropriate interventions to address these health risks.

## 2. Materials and Methods

### 2.1. The Study Areas

**Figure 1:**
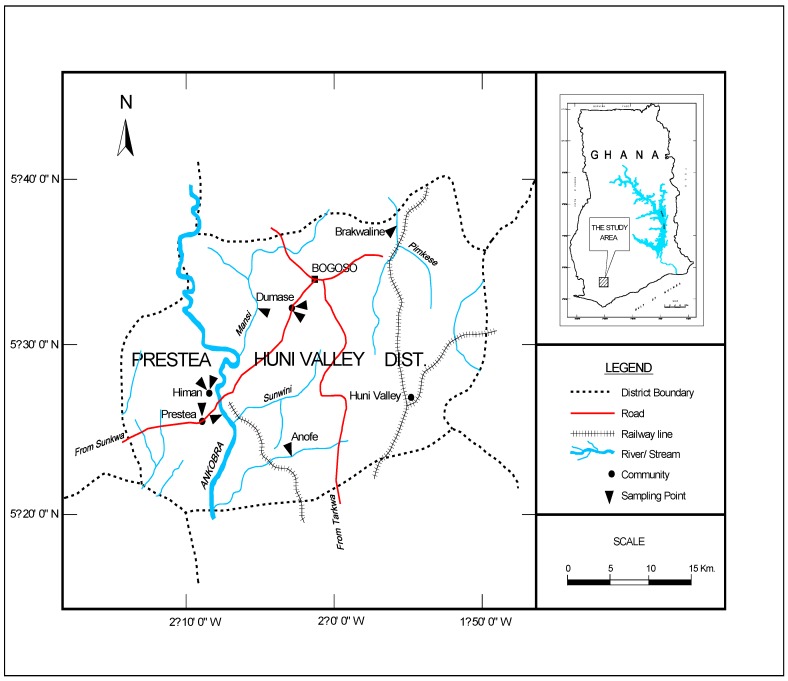
Map of the study area.

There are several mining communities in Ghana; however, this study focuses on the PresteaHuni Valley District, which is considered the hub of mining operations in Ghana. This area hosts four (4) major mining companies, and a sizeable number of artisanal mining companies as well as illegal miners popularly called “*galamsey* operators”. The population of PresteaHuni Valley District is approximately 40,100 [16] and is mainly composed of the indigenous Wassa tribe as well as other tribal entities in Ghana. Subsistence farming is the main occupation of the people, although rubber, palm oil and cocoa are also cultivated. However, in recent times, mining has become the main industrial activity in the area [17]. The area lies within the main gold belt of Ghana that stretches from Axim in the southwest, to Nangodi in the northeast (study area is shown in Figure 1).

### 2.2. Collection of Samples

Random sampling techniques were adopted in selecting nine sampling sites as outlined by the American Water Works Association [18]. The samples were collected on monthly basis between January and May 2011. In all, 100 samples comprising 70 water and 30 sediment samples were collected from nine locations (Figure 1) within this period. The sample containers were washed with detergent and rinsed with 1:1 nitric acid and distilled water. One and half liter (1.5 L) water samples were collected in sampling bottles from each sampling point.

During sampling, the containers were rinsed several times with the sample before the final sample was taken. The water samples were acidified by adding 1 mL of 10% analytical grade nitric acid, stored in an ice chest at 4 °C and was conveyed to the Council for Scientific and Industrial Research—Water Research Institute Heavy Metal laboratory for analysis (the samples were analyzed within a week after collection). The sediments were collected into labelled, pre-washed plastic containers using a Teflon coated soil auger 30 cm from the beds of the rivers/streams from each sampling point. Both samples (water and sediment) collected for each month were analyzed separately for Cd, As, Hg and Pb and the mean as well as the standard deviation of results for Pb, Cd, As and Hg were computed at the end of study [18].

### 2.3. Digestion and Analysis of Selected Toxicants in the Samples

In the laboratory, the acidified water samples were filtered through 0.45 μm Whatman filter paper. For the determination of As and Cd, 5 mL each of concentrated H_2_SO_4_ and concentrated HNO_3_ were added to acidified filtered water samples (100 mL). In the case of Pb, concentrated HNO_3_ (5 mL) was added to 100 mL of the collected water sample. The mixtures were heated on a hot plate until the volume was reduced to about 15–20 mL. The digested samples were allowed to cool to room temperature and then filtered through 0.45 μm Whatman filter paper. The final volume was adjusted to 100 mL with distilled water and the samples stored for analysis [18]. In the case of Hg, to 100 mL of the sample, concentrated H_2_SO_4_ (5 mL) and concentrated HNO_3_ (2.5 mL) were added, followed by the addition of 5% (w/w) KMnO_4_ (15 mL). The mixture was heated in a water bath for 2 h at 95 °C. It was then removed and allowed to cool to room temperature, before 12% (w/w) hydroxylamine hydrochloride (6 mL) was added to reduce the excess permanganate and the mixture was filtered through a 0.45 μm filter paper into a 50 mL volumetric flask and stored for analysis [18].

Blank solutions were prepared for each of the metals analyzed in this study. The blank solution for each metal was analyzed first before the analysis of the samples for that particular toxicant. The source of the blank solution for each particular toxicant in this study was all the reagents used in the digestion process minus the sample; the blanks were prepared in the same way as the samples. The concentration of the metal in the blank solution was automatically stored by the Agilent AAS model number 200 series, instrument coupled with a Graphite Tube Atomizer GTA 120. The blank concentration was then subtracted from the concentration of the metal in the sample to give the actual concentration of the particular metal.

The concentrations of Cd, Pb, and Hg in the digested samples were analyzed after a blank solution for each of the above metals as well as standard solutions of Cd, Pb, and Hg (of concentrations 0.5 mg/L, 1.0 mg/L, 1.5 mg/L, and 2.0 mg/L, respectively, for the aforementioned metals) were used to calibrate the AAS. For the determination of As ions in the samples, a blank solution and standard As solution of concentrations 0.5, 1.0, 1.5, and 2.0 mg/L were used to calibrate the air-acetylene Agilent AAS coupled to an arsine gas generator. In the arsine generator, 0.4% NaBH_4_ (4 mL), and 0.5 M HCl (5 mL) were added to reduce As ions in each sample to arsine gas (AsH_3_). The generated arsine gas was then carried by an argon gas flow to the air-acetylene flame for the determination of As concentration.

### 2.4. Quality Control

The sensitivity of methods used in analysis of the metals was determined using recovery and reproducibility studies, which were conducted using certified standard reference solutions for As, Cd, Pb and Hg manufactured by BDH Chemicals (London, UK). The percentages of As, Pb, and Hg recovered in the recovery studies ranged between 95% and 100% for As, Cd, Pb and Hg, respectively. Similar results were obtained for the reproducibility studies. The percentages of the six toxicants recovered in the reproducibility studies ranged from 97.3% to 98.6% (standard error ±0.02–0.56). In between the analysis, certified reference materials of the aforementioned toxicants were analyzed to verify the calibration curve.

### 2.5. Human Health Risk Assessment

In this study, human health risk assessment is defined as the process of estimating the magnitude of adverse health effects on human exposures to environmental hazards. Risk assessment consists of four fundamental steps namely, hazard identification, exposure assessment, dose-response assessment and risk characterization [19,20]. According to [21,22], the hazard identification process involves review of key research to identify any potential health problems that the metals and the metalloids can cause. Exposure assessment involves the determination of the amount, duration, and pattern of exposure to the heavy metals. Dose-response assessment estimates how much of the metals and metalloids it would take to cause varying degrees of health effects that could lead to illnesses. Finally, risk characterization involves the assessment of the risk for the heavy metals to cause cancer or other illnesses in the population of interest. In this study, the population of interest is artisanal small-scale gold miners in the Prestea Huni Valley District.

#### 2.5.1. Exposure Assessment

The exposure scenario evaluated in this study is an occupational setting involving adult ASGM mine workers aged 18 years and above (and not children below the ages of 18 years who work at ASGM mine sites in the study area). The exposure routes evaluated in this study are oral and dermal routes for the contaminants in sediments or water. Ingestion and dermal contact with sediment and water were considered as appropriate exposure routes through which ASGM miners in the study are exposed to As, Cd, Pb and Hg. This is due to the fact that, the ASGM workers carry out their activities (washing of the gold ores or dredge the water bodies for the alluvial gold) in highly turbid water bodies often with or without protective clothes.

Thus, this study evaluated non-carcinogenic health hazard which refers to harm done to the central nervous system as well as other adverse health effects (except cancer) due to exposure to the aforementioned metals and metalloids of interest; the converse holds for carcinogenic health effects based on both Central Tendency Exposure (CTE) and Reasonable Maximum Exposure (RME) parameters respectively. The CTE parameters as used in this study, assumes that ASGM workers are exposed to fifty percent (50%) of the mean concentrations of As, Cd, Hg and Pb presented in Table 2 and Table 3 below; while the RME parameters assumed that the ASGM workers are exposed to ninety-five (95%) of the mean concentrations of the aforementioned metals and metalloids in Table 2 and Table 3 in Section 3.1 below.

*Ingestion of sediment*: ASGM miners working outdoors in contaminated water bodies may ingest sediment through incidental contact of the mouth with hands and clothing. In this study, intake of As, Cd, Pb and Hg in ingested sediments by ASGM miners was expressed as average daily dose (ADD) which was calculated as follows:

ADD = [(EPC × IR × AAF_so_ × ED × EF × 10^−6^)/(BW × AT)] (1)

where: ADD = Average daily dose, mg/kg-day; EPC = Exposure Point Concentration, *i.e.*, concentration of As, Cd, Pb and Hg in sediment (mg/kg) to be impacted upon by ASGM miners.

IR = Sediment ingestion rate (mg/day); AAF_so_ = Oral-sediment absorption adjustment factor (mg/mg); ED = Exposure Duration (years), *i.e.*, it is assumed that the ASGM miners works on the average 8 h a day for CTE parameters and 12 h a day for the RME parameters; EF = Exposure Frequency (events/year); BW = Body weight (kg); AT = Averaging Time, which is equal to the life expectancy of a resident Ghanaian. With the exception of EPC, ED and BW, the rest were default values in the Risk Integrated Software for clean-up of hazardous waste sites (RISC 4.02) developed by BP for the Superfund sites [23] (U.S. EPA does not endorse the use of this software or others). Bodyweight of 58.6 kg was used for resident adults, in line with data from the Ghana Statistical Service [16]. The average life expectancy for resident adult in Ghana is 65.8 years (*i.e.*, 63.4 years for men and 68.2 years for women) [16], however in this study, an average lifetime expectancy of 70 years was used as life expectancy for ASGM miners in the Prestea Huni Valley District so as to be consistent with [24].

*Dermal contact with sediment*: For ASGM miners in the study area, some sediment contaminants may be absorbed across the skin into the bloodstream. The absorption will take place depending on the amount of sediment in contact with the skin, the concentration of chemicals in sediment, the skin surface area exposed, and the potential for the chemical to be absorbed across skin.

**Table 1:** Intake (default) parameters for adult ASGM miners used in the human health risk assessment from the RISC 4.02 software [23,25].

Parameter	Central Tendency Exposure (CTE)				Reasonable Maximum Exposure (RME)			
	As	Cd	Pb	Hg	As	Cd	Pb	Hg
Averaging Time (Life time)/years	70	70	70	70	70	70	70	70
Body Weight/kg	58.6	58.6	58.6	58.6	58.6	58.6	58.6	58.6
Oral Exposure Frequency for sediment (events/year)	40	40	40	40	350	350	350	350
Dermal Exposure Duration for sediment (year)	9	9	9	9	30	30	30	30
Oral Exposure Frequency for water (events/year)	40	40	40	40	350	350	350	350
Oral Exposure Duration for water (year)	9	9	9	9	30	30	30	30
Ingestion rate for water (L/day)—IR_wo_	2	2	2	2	6	6	6	6
Ingestion rate for sediment (mg/day)—IR_sed_	40	40	40	40	100	100	100	100
Total skin surface area (cm^2^)—SA	18,400	18,400	18,400	18,400	23,000	23,000	23,000	23,000
Fraction of skin exposed to sediment (unitless)	0.11	0.11	0.11	0.11	0.25	0.25	0.25	0.25
Sediment-skin adherence factor (mg/cm^2^)	0.2	0.2	0.2	0.2	0.2	0.2	0.2	0.2
Oral cancer slope factor (1/mg/kg—day)	1.5	0.014	-	-	1.5	0.014	-	-
Dermal cancer slope factor (1/mg/kg—day)	1.5	0.014	-	-	1.5	0.014	-	-
Oral reference dose (mg/kg—day)	0.0003	0.0005	0.0036	0.0003	0.0003	0.0005	0.0036	0.0003
Dermal reference dose (mg/kg—day)	0.0003	0.0005	0.0036	0.0003	0.0003	0.0005	0.0036	0.0003
Oral-sediment absorption adjustment factor (unit less)	1	1	1	1	1	1	1	1
Dermal-sediment absorption adjustment factor (unit less)	0.03	0.001	0.01	0.1	0.03	0.001	0.01	0.1
Oral-water absorption adjustment factor (unit less)	1	1	1	1	1	1	1	1
Dermal-water absorption adjustment factor (unit less)	1	1	1	1	1	1	1	1
Skin permeability coefficient (cm/hr)	0.001	0.001	−	0.0017	0.001	0.001	−	0.0017

According to the USEPA [26,27], Equation (2) is used in calculating the average daily dose of the contaminants in the sediment via dermal contact:

ADD = [(EPC × SA × AAF_sd_ × AF × FS × EF × ED × 10^−6^) / (BW × AT)] (2)

where SA = Total skin surface area exposed to the sediment (cm^2^); AAF_sd_ = Dermal-sediment absorption adjustment factor (mg/mg); AF = Sediment-to-skin adherence factor (mg/cm^2^/event). FS = Fraction of skin area exposed to the sediment (unitless). The other variables were defined in Equation (1) above.

*Ingestion of water*: ASGM miners working outdoors in the contaminated water bodies may ingest water through drinking or incidental drinking or contact of the mouth with hands contaminated with such water bodies. In this study, the average daily dose for each of the toxic metals (*i.e.*, As, Pb, Cd and Hg) ingested in the water bodies by the ASGM mine workers was calculated using Equation (3) below:

ADD = [(EPC × IR × AAF_wo_ × EF × ED × 10^−6^)/(BW × AT)] (3)

where EPC = exposure point concentration of a metal in the drinking water (μg/L), IR = water ingestion rate per unit time (L/day); AAF_wo_ = Oral-water adjustment factor (μg/L). Other variables were defined in Equations (1) and (2) above.

*Dermal contact with water*: For ASGM miners in the study area, the contaminants in the water may be absorbed across the skin into the bloodstream when the miners wash the ore or dredge for alluvial ores from the stream/river. The absorption will take place depending on the volume of water in contact with the skin, the concentration of chemicals in the water, the skin surface area exposed, and the potential for the chemical to be absorbed across skin. Equation (4) as published by USEPA [28], was used in calculating the uptake of the toxicants in water bodies via dermal exposure pathway in this study:

ADD = [(EPC × K_p_ × SA × AAF_wd_ × EF × ED × 10^−6^)/(BW × AT)] (4)

where all the symbols have the same meaning as in Equation (3) above, with the exception K_p_ and AAF_wd_. K_p_ = Skin permeability coefficient (cm/h); AAF_wd_ = Dermal-water absorption adjustment factor (μg/L).

The skin permeability coefficient (K_p_) estimates the permeability of the aforementioned toxicants through the skin layers into the blood stream of ASGM miners in the study area, default values from the RISC 4.02 software were used for each of the toxicants in this study.

Default values from the Risk Integrated Software for clean-up of hazardous waste sites (RISC 4.02) were used for the following parameters in Equations (2)–(4) above; IR, AAF_wo_, AAF_wd_, AAF_so_, AAF_sd_, SA, EF and K_p_ for each toxicant and this can be found in Table 1 above). The rationale for the use of default values for exposure duration and exposure frequency in Table 1 were based on assumptions outlined by [24,27].

#### 2.5.2. Hazard Identification and Dose-Response Assessment

Metals and metalloids such as Cd, As, Pb and Hg are toxic to humans and produce multiple adverse health effects, even at low concentrations via exposure to them in the environment (either through the food chain or oral, dermal and inhalation from contaminated environmental media such as water, sediment, soil or air). They also have the ability to bioaccumulate in human beings, organisms and in environmental media such as water, soil, sediment or food crops [12]. For example, a strong linkage has been established that long term exposure to cadmium via inhalation route is known to cause lung cancer in human beings [28,29]; however, other exposure routes for cadmium by human beings have been linked to prostate cancer, renal cancer, liver cancer and stomach cancer, although this linkage is very weak [29]. Exposure to lead has adverse effects on the renal and central nervous systems, haematopoiesis; Pb in its inorganic form is classified as a possible human carcinogen by the International Agency for Research on Cancer [30].

According to the World Health Organization (WHO) [12], arsenic has been classified as a class 1 human carcinogen, based on sufficient evidence from human epidemiological data that exposure to arsenic causes several forms of cancers [31,32]. For instance, high cases of lung cancer mortality were observed in multiple human populations exposed to arsenic primarily through inhalation [12]. Also, increased mortality from multiple internal organ cancers (liver, kidney, lung and bladder) and an increased incidence of skin cancer were observed in populations consuming drinking water high in inorganic arsenic [33].

It has been noted that exposure to mercury is known to be a causative agent of neurological, nephrological, cardiac and reproductive disorders, as well as genetic damage and disrupting certain functions of the endocrine system [34]. Within small-scale gold mining communities in Ghana, elevated exposures to the aforementioned metals in miners and community members is now being documented via biomonitoring studies [35,36]. Dose-response assessment describes the degree or incidence or likelihood of hazard associated with a particular dose of contaminant (*i.e.*, As, Cd, Hg and Pb).

#### 2.5.3. Calculation of Carcinogenic and Non-Carcinogenic Health Risk

The likelihood or incidence of hazard estimated for ASGM miners under different exposure scenarios routes is part of the risk characterization of this population. In order to assess the carcinogenic and non-carcinogenic health risk of ASGM miners from dermal and oral exposure through water and sediments from the study area, the average daily dose calculated from Equations (1)–(5) above were used. As is recommended in U.S. EPA guidance documents [26], the non-carcinogenic health risk faced by ASGM miners from exposure to the toxicants in the water and sediment samples via oral and dermal contact based on both CTE and RME parameters are expressed in terms of the hazard quotient (HQ).

The hazard quotient for an individual chemical and individual exposure route is calculated by:

HQ = ADD/RfD(5)

where ADD = the average daily dose ASGM miner is exposed to As, Pb, Cd and Hg in water and sediment via oral and dermal exposure routes respectively (mg/kg—day); RfD = the reference dose for each metal or metalloid for each exposure route. The RfD is an estimate (with uncertainty spanning perhaps an order of magnitude) of a daily oral or dermal exposure to the human population (including sensitive sub-groups) that is likely to be without an appreciable risk of deleterious effects during a lifetime [26].

According to the US EPA Exposure Factors Handbook, where the oral absorption of toxicants in water is about 95%, there is no need to adjust the oral reference dose values [26]. Since oral absorption of the toxicants measured in this study in water ≥95%, then there was no need to adjust the oral reference dose values for each toxicant [26].

Cancer risk estimates by ASGM miners from oral and dermal contact of As and Cd in the water and sediment samples from the study area were calculated using Equation (6) below:

Cancer Health Risk = (ADD) × CSF(6)

where ADD = the average daily dose of As or Cd in water and sediment via oral and dermal exposure routes in the study area by ASGM miners; CSF = cancer slope for each metal or metalloid and for each exposure route. The cancer slope factor (CSF) unlike the reference dose for non-carcinogenic health risk assumes that exposure to any amount of a carcinogen will increase the risk of cancer, *i.e.*, there is no safe or threshold dosage [26]. A cancer slope factor is an upper bound, approximating a 95% confidence limit, on the increased cancer risk from a lifetime exposure to toxicant by ingestion, dermal or inhalation exposure route [26].

In this study, the oral and dermal reference doses for the respective toxicants used including the cancer slope factors as well as other intake default parameters from the RISC 4.02 software which were used in Equations (1)–(4) above are presented in Table 1 above.

## 3. Results and Discussion

### 3.1. Summary Statistics of Physicochemical Parameters and Heavy Metals

Table 2 and Table 4 present the mean levels of physico-chemical parameters and heavy metals; while the mean concentration of heavy metals in the sediment is presented in Table 3. From Table 4, the mean pH value of the water samples is slightly acidic, with the values ranging from 5.75 to 6.97 pH units.

The mean value of turbidity for all locations did not meet the GS 175/1-WHO Guideline value of 5 NTU [37]. From the results of TDS presented in Table 4, TDS for the entire sample locations were within GS 175/1-WHO guideline value [37]. According to [37], there is no adverse health effects associated with exposure to drinking water with TDS below 1000 mg/L. Drinking water becomes significantly and increasingly unpalatable to consumers if the TDS value exceeds 1000 mg/L. As, noted by [38], if the TDS of drinking water exceeds 1000 mg/L, it may pose significant health challenges to consumers, particularly those that are hypertensive, diabetic, or undergoing renal dialysis.

**Table 2:** Mean levels of metals and metalloids in water samples in the study areas.

Sampling Station (n)	Community	As—μg/L		Cd—μg/L		Pb—μg/L		Hg—μg/L	
		* Mean (SD)	Min–Max	* Mean (SD)	Min–Max	* Mean (SD)	Min–Max	* Mean (SD)	Min–Max
River Anikoko	Brakwaline	325 *(3.54**)**	320–330	230 **(19.5)	203–258	0.20 (0.007)	0.19–0.21	154 **(24.4)	120–189
River Abodwesh	Himan	25 ** (5.79)	18–34	340 **(17.6)	321.2–368.2	0.44 (0.09)	0.30–0.58	178 **(36.6)	132–234
River Ankobra	Prestea	45 **(9.35)	30–55	30 **(5.17)	23.0–37.6	0.21 (0.04)	0.16–0.28	561 **(87.0)	438–684
River Amenkume	Prestea	103 ** (7.07)	93–113	45 **(2.83)	41–49	0.19 (0.04)	0.14–0.24	246 **(66.1)	153–340
River Dinyame	Himan	24 ** (4.64)	18–31	0.21 (0.05)	0.14–0.29	0.36 (0.14)	0.19–0.58	866 ** (20.2)	836–893
River Anfoe	Anofe	42 ** (8.49)	30–54	4.00 ** (0.37)	3.6–4.6	122 ** (19.2)	99–152	278 ** (56.6)	198–358
River Woawora	Dumase	112 ** (14.2)	98–135	5.00 ** (0.72)	3.9–5.9	33 ** (7.41)	25.7–45.0	321 ** (10.6)	306–336
River Benyan	Dumase	15 ** (4.95)	8–22	61.0 ** (6.28)	51–68	0.22 (0.08)	0.11–0.35	472 ** (14.1)	452–492
River Mansi	Dumase	21 ** (5.09)	13.8–28.2	0.17 (0.02)	0.14–0.20	0.17 (0.02)	0.14–0.20	132 ** (1.22)	131–134
GSA—175/WHO	−	10		3		10		10	

Notes: * The concentrations reported in this table are the means; SD represents standard deviation. ** Bold figures exceeded GS 175-1/WHO permissible guideline values [37].

**Table 3:** Mean levels of metals and metalloids in sediment samples in the study areas.

Sampling Station	Community	As mg/kg		Cd—mg/kg		Pb—mg/kg		Hg—mg/kg	
		* Mean (SD	Min–Max	* Mean (SD)	Min–Max	* Mean (SD)	Min–Max	* Mean (SD)	Min–Max
River Anikoko	Brakwaline	0.987 (0.081)	0.871**–**1.102	0.162 (0.016)	0.139**–**0.185	0.008 (0.003)	0.004**–**0.012	0.469 (0.143)	0.267**–**0.671
River Abodwesh	Himan	0.605 (0.051)	0.532**–**0.678	0.067 (0.019)	0.041**–**0.094	0.010 (0.001)	0.008**–**0.012	0.789 (0.164)	0.557**–**1.021
River Ankobra	Prestea	0.313 (0.069)	0.215**–**0.412	0.092 (0.013)	0.073**–**0.111	0.124 (0.009)	0.11**–**0.133	0.562 (0.185)	0.301**–**0.823
River Amenkume	Prestea	0.276 (0.002)	0.273**–**0.279	0.073 (0.016)	0.051**–**0.095	0.005 (0.002)	0.002**–**0.008	0.168 (0.035)	0.118**–**0.218
River Dinyame	Himan	0.063 (0.004)	0.058**–**0.068	0.044 (0.006)	0.035**–**0.053	0.053 (0.013)	0.035**–**0.071	0.801 (0.057)	0.720**–**0.882
River Anfoe	Anofe	0.041 (0.004)	0.036**–**0.046	0.063 (0.022)	0.032**–**0.094	0.034 (0.008)	0.022**–**0.046	0.130 (0.014)	0.110**–**0.150
River Woawora	Dumase	0.032 (0.004)	0.027**–**0.037	0.053 (0.013)	0.035**–**0.071	0.023 (0.006)	0.014**–**0.032	0.231 (0.023)	0.199**–**0.263
River Benyan	Dumase	0.021(0.002)	0.018**–**0.024	0.023(0.006)	0.014**–**0.032	0.042 (0.011)	0.026**–**0.058	0.140 (0.007)	0.130**–**0.150
River Mansi	Dumase	0.102 (0.012)	0.085**–**0.119	0.015 (0.004)	0.009**–**0.021	0.172 (0.017)	0.148**–**0.196	0.120 (0.007)	0.110**–**0.130
GSA—175/WHO [39]	−	27		6		−		−	

Notes: * The concentrations reported in this table are the means; SD represents standard deviation.

All heavy metal and metalloid concentrations measured in water samples from this study were observed to be above the GS 175/1-WHO recommended guideline values, except for cadmium in Rivers Dinyame and Mansi, where they were found to be below the GS 175/1-WHO guideline values (Table 2).

**Table 4:** Physical water quality parameters of water samples from the study areas.

Sampling Station	Community	Parameters				
		pH (pH Units)	Conductivity (μS/cm)	Turbidity (NTU-Nephelometric Turbidity Unit)	Total Dissolved Solids (mg/L)	** Total Suspended Solids (mg/L)
River Anikoko	Brakwaline	5.75 *	127	1000 *	69.9	30,330
River Abodwesh	Himan	6.46 *	180	1000 *	99.0	18,300
River Ankobra	Prestea	6.78	152	1000*	83.6	10,500
River Amenkume	Prestea	6.59	136	1000 *	74.8	4667
River Dinyame	Himan	6.62	241	1000 *	133	47,400
River Anfoe	Anofe	6.91	156	1000 *	85.8	8800
River Woawora	Dumase	6.97	345	35.1 *	190	600
River Benyan	Dumase	6.34 *	82.8	1000 *	45.5	15,400
River Mansi	Dumase	6.02 *	201	584 *	36.5	1500
GSA—175/WHO	−	6.5–8.5	−	**5**	1000	−

Notes: * Bold figures exceeded GS 175—1/WHO permissible guideline values [37]. ** TSS = means total suspended solids.

A similar study conducted in the Kibi traditional area in Ghana to determine mean levels of As, Cd, Hg and Pb in water samples reported that mean lead values of 25 μg/L (Obronikrom), 18 μg/L(Kibi-Deaf), 6 μg/L each (Bunso and Apapam); while in the case of arsenic, the mean As values were 180 mg/L (Obronikrom) and 46 mg/L each (Kibi-Deaf and Bunso) [40]. The mean levels of Hg and Cd reported in this study are comparable to those reported in studies of other artisanal gold mine sites in Ghana [41,42].

It can be seen from Table 3 that the mean levels of As, Cd, Hg and Pb in sediments from the rivers sampled in this study were found to be below the acceptable guideline values. This observation is due to continuous ASGM activities such as dredging of the rivers for alluvial gold does not allow these metals and metalloids to settle into the sediments.

The pH and TDS are a major concern in this study since they are the vital factors in metal and metalloid solubility and control their speciation and thus their distribution within dissolved fractions [43]. This has some implications for the potential health risk faced by ASGM miners who work in such water bodies, because it may influence the amount of As, Cd, Hg or Pb in either the water or the sediment that can be impacted upon by ASGM workers.

### 3.2. Cancer Risk Results

The results of cancer risk and non–cancer health hazards for resident adults from the study area exposed to As, Cd, Hg and Pb via oral ingestion of water are presented in Table 5. Cancer health risk is defined as the incremental probability that an artisanal miner would develop cancer during his or her lifetime due to chemical exposure under specific exposure scenarios evaluated. According to U.S. EPA Exposure Factors and the International Agency Research on Cancer (IARC), As and Cd are the two metals among the list of metals measured in this study that are likely to be carcinogenic via oral ingestion and dermal contact with contaminated water bodies or sediments; hence, the calculation of their carcinogenic risk in this study (Table 5) [26,33]. From Table 5, the estimated lifetime cancer risk results for artisanal miners working along River Mansi in Dumase is 3.1 × 10^−3^ and 1.3 × 10^−3^ using CTE parameters for oral and dermal contact of arsenic in the contaminated water from River Mansi.

Additionally, the cancer health risk for artisanal mine workers working in and around River Worawora who are exposed to As in water samples from River Worawora is 4.0 ×10^−2^, and 3.1× 10^−2^ for oral ingestion route based on CTE and RME parameters, respectively.

The evaluation of cancer risks from exposure to As and Cd in most of the water samples in this study by artisanal mine workers were found to be above the acceptable U.S. EPA cancer health risk range of 1.0 × 10^−6^ to 1 × 10^−4^ (*i.e.*, 1 case of cancer per every 1,000,000 to 1 case of cancer per every 10,000), while in the case of the sediments most of the cancer risks faced by artisanal mine workers were found to be within the acceptable U.S. EPA ranges [44].

### 3.3. Non-Cancer Health Hazard Results

A HQ greater than 1.0 is generally interpreted as a level of concern [30,45]. Non-carcinogenic health hazards arising from exposure to Pb, Cd, Hg and As for artisanal mine workers in the CTE and RME for oral ingestion and dermal contact in water samples are shown in Table 6, while Table 7 displays the results for oral ingestion and dermal contact with the toxicants of concern in sediment by artisanal miners from this study via CTE and RME scenarios, respectively.

A comparison of Table 6 and Table 7 indicates that dermal HQs from water (Table 6) for As, Pb and Hg exceeded the HQ guide value of 1.0 for all the sampling points; in the case of Cd, 11.1% of all the sampling point in Table 6 exceeding HQ value of 1.0 as against all HQ values for Cd in Table 7 below HQ < 1.0.

This indicates that dermal exposure to water from the study area cannot be disregarded as a source of health concerns. An examination of HQs from dermal water contact (Table 6) indicates that the RME scenario generally presents HQs greater than the CTE scenario. For As, Cd, Pb and Hg, the following RME scenarios exceeds an HQ value of 1.0 (*i.e*., >1) for As (in all the nine sites), Cd (two sites), Pb (nine sites) and Hg (nine sites). Given the fact that HQ values for As were recorded in this study, the ASGM miners may be susceptible to non-cancer diseases that have been identified by other workers [19,46,47,48,49,50]; while the documented disease associated with mercury exposure via oral or dermal contact to water by ASGM workers are as follows, it has teratogenic effects, damages the renal, nervous, gastrointestinal tract and the respiratory system [19,20,51]; in the case of cadmium, other symptoms associated with cadmium exposure include irritation of the upper respiratory tract, metallic taste in the mouth, cough and chest pains [52].

Ingestion of elevated levels of cadmium has resulted in toxicity to the kidney and skeletal systems and may be associated with an elevated incidence of hypertension and cardiovascular disease [53]. From Table 6 below, it can be seen that the hazard quotient values for Pb via oral and dermal contact with the contaminated water by ASGM miners in this study exceeded the guideline value of 1.0; which suggests that the ASGM miners are likely to suffer from diseases such as stroke, heart attack, haematopoiesis, *etc.*

**Table 5:** Cancer health risk results from exposure to arsenic and cadmium by artisanal mine workers in the Prestea Huni Valley District.

Sampling Point	Communities	Exposure Medium	Exposure Route	Cancer Risk			
				Arsenic		Cadmium	
				CTE	RME	CTE	RME
River Anikoko	Brakwaline	Sediment	Oral	3.2 × 10^−5^	6.2 × 10^−4^	8.4 × 10^−6^	6.1 × 10^−4^
			Dermal	1.9 × 10^−6^	7.6 × 10^−4^	2.1 × 10^−6^	2.1 × 10^−4^
		Water	Oral	4.3 × 10^−3^	1.3 × 10^−2^	2.4 × 10^−3^	7.1 × 10^−2^
			Dermal	3.0 × 10^−3^	4.0 × 10^−3^	4.3 × 10^−3^	3.2 × 10^−2^
River Abodwesh	Himan	Sediment	Oral	4.3 × 10^−4^	1.3 × 10^−2^	7.6 × 10^−6^	5.2 × 10^−4^
			Dermal	8.0 × 10^−4^	6.0 × 10^−3^	2.1 × 10^−6^	1.8 × 10^−4^
		Water	Oral	1.6 × 10^−3^	4.9 × 10^−2^	2.2 × 10^−3^	6.6 × 10^−2^
			Dermal	3.0 × 10^−3^	2.3 × 10^−2^	4.1 × 10^−3^	3.1 × 10^−2^
River Amenkume	Prestea	Sediment	Oral	1.3 × 10^−5^	9.3 × 10^−4^	2.8 × 10^−6^	6.8 × 10^−2^
			Dermal	3.9 × 10^−6^	3.3 × 10^−4^	8.4 × 10^−7^	3.1 × 10^−2^
		Water	Oral	4.7 × 10^−4^	6.7 × 10^−4^	7.7 × 10^−4^	6.5 × 10^−3^
			Dermal	3.2 × 10^−5^	5.1 × 10^−4^	4.5 × 10^4^	7.5 × 10^−3^
River Ankobra	Prestea	Sediment	Oral	7.6 × 10^−4^	2.7 × 10^−4^	9.6 × 10^−3^	9.8 × 10^−3^
			Dermal	5.2 × 10^−4^	4.0 × 10^−4^	7.6 × 10^−5^	7.6 × 10^−4^
		Water	Oral	1.4 × 10^−4^	2.3 × 10^−3^	1.9 × 10^−3^	1.3 × 10^−2^
			Dermal	4.1 × 10^−4^	1.4 × 10^−3^	2.3 × 10^−3^	1.2 × 10^−2^
River Dinyame	Himan	Sediment	Oral	3.6 × 10^−4^	2.4 × 10^−4^	1.8 × 10^−3^	1.8 × 10^−2^
			Dermal	1.0 × 10^−4^	8.4 × 10^−3^	1.6 × 10^−3^	1.5 × 10^−2^
		Water	Oral	6.0 × 10^−3^	5.3 × 10^−3^	8.4 × 10^−2^	3.3 × 10^−2^
			Dermal	7.1 × 10^−3^	1.8 × 10^−3^	6.7 × 10^−3^	6.1 × 10^−2^
River Anfoe	Anofe	Sediment	Oral	7.6 × 10^−5^	2.4 × 10^−4^	1.9 × 10^−5^	2.9 × 10^−2^
			Dermal	5.3 × 10^−4^	3.2 × 10^−5^	5.6 × 10^−3^	2.3 × 10^−2^
		Water	Oral	2.6 × 10^−3^	7.7 × 10^−2^	1.9 × 10^−3^	1.3 × 10^−2^
			Dermal	4.7 × 10^−3^	3.5 × 10^−2^	2.3 × 10^−3^	1.2 × 10^−2^
River Woawora	Dumase	Sediment	Oral	3.2 × 10^−3^	2.5 × 10^−3^	9.0 × 10^−2^	7.8 × 10^−2^
			Dermal	9.8 × 10^−3^	8.1 × 10^−3^	7.2 × 10^−2^	5.6 × 10^−2^
		Water	Oral	4.0 × 10^−2^	3.1 × 10^−2^	8.8 × 10^−2^	7.5 × 10^−2^
			Dermal	4.9 × 10^−2^	1.4 × 10^−2^	5.8 × 10^−2^	5.3 × 10^−2^
River Benyan	Dumase	Sediment	Oral	4.3 × 10^−6^	6.1 × 10^−4^	2.8 × 10^−2^	7.5 × 10^−2^
			Dermal	2.3 × 10^−3^	1.1 × 10^−4^	5.4 × 10^−3^	8.4 × 10^−3^
		Water	Oral	2.3 × 10^−3^	6.9 × 10^−2^	4.5 × 10^−2^	8.3 × 10^−2^
			Dermal	4.2 × 10^−3^	3.2 × 10^−2^	6.2 × 10^−2^	7.3 × 10^−2^
River Mansi	Dumase	Sediment	Oral	3.8 × 10^−3^	5.9 × 10^−2^	2.3 × 10^−5^	3.7 × 10^−4^
			Dermal	1.3 × 10^−3^	4.7 × 10^−2^	7.1 × 10^−6^	2.9 × 10^−4^
		Water	Oral	3.1 × 10^−3^	4.3 × 10^−2^	4.5 × 10^−2^	7.4 × 10^−2^
			Dermal	1.3 × 10^−3^	9.9 × 10^−2^	5.6 × 10^−3^	5.9 × 10^−3^

**Table 6:** Non-cancer health hazard faced by artisanal mine workers from exposure to As, Cd, Pb and Hg via oral ingestion and dermal contact water from rivers in the study area.

Sampling Station	Location	Exposure Route	Non-Cancer Health Hazard (Hazard Quotient)							
			As		Cd		Pb		Hg	
			CTE	RME	CTE	RME	CTE	RME	CTE	RME
**River Anikoko**	Brakwaline	Oral	1.98	1.78	0.04	0.06	1.45	1.40	4.60	4.50
		Dermal	1.20	34.0	0.05	1.10	1.34	1.10	1.23	1.15
**River Abodwesh**	Himan	Oral	0.97	1.90	0.04	0.09	1.25	1.67	1.23	1.19
		Dermal	1.40	1.14	0.05	0.10	1.83	2.15	2.23	3.15
**River Amenkume**	Prestea	Oral	1.02	1.71	1.02	1.71	1.43	1.78	1.13	1.10
		Dermal	1.17	6.30	1.07	1.63	1.38	1.79	1.12	1.91
**River Ankobra**	Prestea	Oral	2.70	99.0	0.27	0.99	6.00	11.0	1.9	14.0
		Dermal	1.90	67.0	0.25	0.50	6.00	1.20	4.1	7.30
**River Dinyame**	Dinyame	Oral	1.30	2.00	0.07	0.15	1.12	1.21	1.12	1.21
		Dermal	1.27	9.80	0.04	0.11	1.60	1.10	1.60	1.62
**River Anfoe**	Anofe	Oral	1.23	8.30	0.03	0.62	1.35	1.72	1.51	1.90
		Dermal	2.34	12.0	0.46	0.92	1.75	1.30	1.75	1.30
**River Woawora**	Dumase	Oral	2.30	3.00	0.20	0.40	1.33	1.58	1.10	1.58
		Dermal	1.04	1.14	0.18	0.65	1.14	1.50	1.12	1.95
**River Benyan**	Dumase	Oral	1.27	9.80	0.04	0.11	1.60	2.10	1.40	1.62
		Dermal	1.23	8.30	0.03	0.62	1.35	2.72	0.51	0.90
**River Mansi**	Dumase	Oral	1.34	12.0	0.46	0.92	1.75	2.30	1.75	3.30
		Dermal	2.30	3.00	0.20	0.40	1.33	1.58	2.10	4.58

**Table 7:** Non-cancer health hazards faced by artisanal mine workers from exposure to As, Cd, Pb and Hg via oral ingestion and dermal contact of sediments from rivers in the study area.

Sampling Station	Location	Exposure Media	Non-Cancer Health Hazard (Hazard Quotient)							
			As		Cd		Pb		Hg	
			CTE	RME	CTE	RME	CTE	RME	CTE	RME
**River Anikoko**	Brakwaline	Oral	0.013	0.029	0.0002	0.005	0.032	0.098	0.026	0.042
		Dermal	0.980	1.240	0.005	0.010	0.011	0.025	0.12	0.21
**River Abodwesh**	Himan	Oral	0.007	0.021	0.0009	0.024	0.015	0.019	0.019	0.028
		Dermal	0.032	0.420	0.015	0.051	0.28	0.835	0.25	0.67
**River Amenkume**	Prestea	Oral	0.039	0.890	0.0021	0.071	0.045	0.88	0.010	0.045
		Dermal	0.017	0.063	0.0062	0.084	0.031	0.079	0.02	0.051
**River Ankobra**	Prestea	Oral	0.120	0.490	0.012	0.199	0.061	0.18	0.021	0.014
		Dermal	0.220	0.620	0.0025	0.054	0.071	0.088	0.002	0.072
**River Dinyame**	Himan	Oral	0.130	0.210	0.007	0.015	0.021	0.041	0.004	0.070
		Dermal	0.022	0.025	0.037	0.112	0.061	0.152	0.023	0.051
**River Anofe**	Anofe	Oral	0.042	0.814	0.0021	0.031	0.24	0.48	0.090	0.54
		Dermal	0.350	0.460	0.012	0.042	0.031	0.062	0.010	0.58
**River Worawora**	Dumase	Oral	0.037	0.260	0.0051	0.085	0.75	0.92	0.011	0.078
		Dermal	0.076	0.980	0.011	0.21	0.074	0.251	0.073	0.094
**River Benyan**	Dumase	Oral	0.220	0.620	0.0025	0.054	0.071	0.088	0.002	0.072
		Dermal	0.130	0.210	0.007	0.015	0.021	0.041	0.004	0.070
**River mansi**	Dumase	Oral	0.022	0.025	0.037	0.112	0.061	0.152	0.023	0.051
		Dermal	0.042	0.814	0.0021	0.031	0.240	0.48	0.090	0.540

As noted in studies by [30,45] strong associations have been established between exposure to lead and human mortality due to stroke and heart attack as well as haematopoiesis [30,45]. A similar study by [54] has been to establish a relationship between exposure to lead and the incidence of nephrotoxicity.

Arsenic-related cancer and non-cancer risk results obtained in this study were higher than the values obtained by [27] for resident adults living in mining communities in Ghana and who are not directly working as ASGM miners.

Risk estimates assumed that ASGM miners spend their lifetime (70 years) exposed to the annual mean concentrations of As, Cd, Pb and Hg (presented in Table 2 and Table 3 above) in water and sediment which should remain constant for the entire 70 years period. This assumption could lead to overestimation of the potential health risk if levels of As, Cd, Pb and Hg in water or sediments decline. However, it could also lead to under estimation if due to increased mining activities leads to an increase in the levels of the aforementioned chemicals in the water or sediment samples. Exposure to As, Cd, Pb and Hg in air or through other media such as food by the ASGM miners were not taken into account. Omission of these exposures may either over or under estimates the potential health risk faced by the ASGM miners in the study area. It was also assumed that exposure duration, exposure frequency as well as other input parameters such as water ingestion rate (*i.e.*, 2 and 6 liters of water per day via CTE and RME parameters) and others in Table 1 above did not change over the 70 years lifetime and that the activities of ASGM miners in the study area remain the same. Notwithstanding these limitations, the findings of this study are relevant and hold several policy implications. As noted by [55,56,57], this type of risk estimates provide ways to screen those pollutants that are of public health concern in order to prioritize research and policy interventions.

## 4. Conclusions

The results of the study revealed artisanal mine workers in the study area are at risk of developing cancerous and other non-cancerous diseases due to exposure to the aforementioned metals. Given low HQ values from dermal contact of the aforementioned toxic chemicals in sediments, it is imperative to undertake an extensive bioavailability studies to determine levels of metals and metalloids in sediments that are constantly been agitated. Also, further work such as case —control epidemiological studies needs to be undertaken to establish a relationship between exposure to As, Cd, Pb and Hg in water and sediment by ASGM miners in Ghana with diseases associated with the aforementioned metals and metalloids identified in studies conducted in other countries. The results from such studies could feed into the development of policy intervention that will protect ASGM mine workers in Ghana.

Given the fact that this study only focused on assessing cancer and non-cancer health risk faced by ASGM mine workers via oral and dermal contact with As, Cd, Hg and Pb in water and sediment, it recommended that other exposure pathways such as inhalation of air or dust by the ASGM workers as well as ingestion of food crops grown in the study area should be considered when undertaking full human health risk assessment ASGM operations in Ghana.

The use of default values in the RISC 4.02 software together with its underlying assumptions for Ghanaian ASGM mine workers may either overestimate or underestimate the cancer and non-cancer health risk. This may introduce some uncertainties in the results obtained in this study. It is within this context that, this study recommended the development of default values or software for undertaking human health risk assessment that will address the peculiarities of Ghanaians. 
